# Changes in life history parameters and transcriptome profile of *Serangium japonicum* associated with feeding on natural prey (*Bemisia tabaci*) and alternate host (*Corcyra cephalonica* eggs)

**DOI:** 10.1186/s12864-023-09182-y

**Published:** 2023-03-14

**Authors:** Shaukat Ali, Jing Peng, Jian-Feng Liang, Chuyang Huang, Yong-Hui Xie, Xingmin Wang

**Affiliations:** 1 Guangdong Laboratory for Lingnan Modern Agriculture，College of Plant Protection, South China Agricultural University, 510642 Guangzhou, P. R. China; 2 Engineering Research Center of Biological Control, Ministry of Education and Guangdong Province, South China Agricultural University, 510642 Guangzhou, China; 3 Kunming Branch of Yunnan Provincial Tobacco Company, 650021 Kunming, China

**Keywords:** *Bemisia tabaci*, *Corcyra cephalonica*, *Serangium japonicum*, Life history, Transcriptome

## Abstract

**Background:**

The mass production of natural predators with prolonged shelf life is a prerequisite for their field application as pest control agents. The traditional methods used for the mass production of *Serangium japonicum* rely heavily on the consistent supply of natural prey. This study explains the effects of *B. tabaci* (natural prey) and *C. cephalonica* eggs (alternative food) on life history and transcriptome profile of *S. japanicum.*

**Methods:**

This study compares the effects of *B. tabaci* (natural prey) and *C. cephalonica* eggs (alternative food) on biology, reproduction, and predatory efficacy, and transcriptome profile of *S. japanicum.*

**Results:**

This study revealed that *S. japonicum* was able to successfully complete its life cycle while feeding on *B. tabaci* (natural prey) and *C. cephalonica* eggs (alternative food). The *C. cephalonica* eggs fed *S. japonicum* individuals had longer developmental period and lower fecundity as compared to those feeding on whitefly but the survival rates (3rd instar nymphs, 4th instar nymphs and pupae) and predatory efficacy of *C. cephalonica* eggs fed *S. japonicum* individuals were significantly similar to to those feeding on whitefly.Transcriptome analysis showed that when faced with dietary changes, *S. japanicum* could successfully feed on *C. cephalonica* eggs by regulating genes related to nutrient transport, metabolism, and detoxification. Moreover, *S. japanicum* degraded excess cellular components through ribosomal autophagy and apoptosis, which provided sufficient materials and energy for survival and basic metabolism.

**Conclusion:**

*Corcyra cephalonica* eggs can be used as an alternate host for the predator, *Serangium japonicum,* as the survival rates and predatory efficacy of the predator are similar to those feeding on the natural host (*B.tabaci)*. When faced with dietary changes, *S. japanicum* could successfully feed on *C. cephalonica* eggs as revealed by upregulation of genes related to nutrient transport, metabolism, and detoxification. These findings are of great significance for studying the functional evolution of *S. japonicum* in response to dietary changes.

**Supplementary Information:**

The online version contains supplementary material available at 10.1186/s12864-023-09182-y.

## Background

Using the natural enemies of insects for pest control is widely considered as a good strategy for reducing the use of chemical pesticides and avoiding their adverse effects. The mass production of natural predators with a prolonged shelf life is a prerequisite for their field application as pest control agents [1, 2]. The traditional methods used for the mass production of insect predators rely heavily on the consistent supply of natural prey. Therefore, changes in prey populations due to environmental factors (such as temperature, photoperiod, and humidity) pose a challenge for the maintenance of the complex three-level nutrient system for the mass production of insect predators [3–5]. Thus, developing methods of mass production of insect predators using cheaper food sources can help promote the application of these biological pest control agents.

Using alternate food sources for the mass production of predatory ladybirds can support their development, but results in longer developmental periods, lower survival rates, and a serious reduction in reproductive potential [6]. Nutrigenomics is a rapidly emerging field of research that focuses on diet-related genomic changes that define nutrient-gene interactions within the host [7, 8]. Zou et al. [9] sequenced the transcriptomes of *Arma chinensis* individuals fed on an artificial diet and an insect prey (pupae of the Chinese oak silk moth, *Antheraea pernyi*). Based on the sequences, the researchers identified several differentially expressed genes (DEGs) associated with changes in various characteristics of *A. chinensis* individuals fed different diets. In *Harmonia axyridis*, the addition of vitellogenin to the artificial diet increased the activity of digestive enzymes and the expression of related genes [10]. Moreover, transcriptome analysis of *Coccinella septempunctata* by Cheng et al. [11] revealed that genes related to amino acid, fat, and starch and glucose metabolism were downregulated in *C. septempunctata* adults fed on artificial diets.

*Serangium japonicum* (Coleoptera; Coccinellidae) is an obligate predator of multiple whitefly species, and can prey on *B. tabaci*, *Aleurocanthus camelliae* and *Dialeurodes citri* [12–15]. *S. japanicum* has shown excellent pest-control of *B. tabaci* under laboratory as well as field conditions [16, 17]. A single *S. japanicum* adult can consume more than 700 *B. tabaci* eggs per day, which can effectively control the population growth of *B. tabaci* [18]. Since the mass production of *S. japanicum* rely heavily on the consistent supply of natural prey (*B. tabaci*), the screening/search of alternate food sources for mass production of *S. japanicum* can be an area of high interest to promote the field application of *S. japanicum.*

*Corcyra cephalonica* is a globally distributed stored grain pest that feeds on rice and wheat bran throughout the year [19]. *Corcyra cephalonica* eggs are widely used for the mass production of different biological control agents. Khuhro et al. [20] reported high survival rates for *Chrysoperla sinica* reared on *C. cephalonica* eggs, thus confirming the higher nutritional quality of *C. cephalonica* eggs. Ding et al. [21] also studied the effects of alternate preys (*C. cephalonica* and *Ephestia kuehniella*) as on development and fecundity of *S. japanicum* by feeding the larval instars of *S. japonicum* on eggs of both species (starting from 1st instar) and concluded that *C. cephalonica* eggs are not suitable for *S. japanicum* rearing. Therefore, this study aims at utilizing *C. cephalonica* eggs as alternative food source for the mass production of *S. japanicum* larvae from 2nd larval instar onwards.

The major objectives of this study were to observe the effects of *B. tabaci* (natural prey) and *C. cephalonica* eggs (alternative food) on biology, reproduction, and predatory efficacy, and transcriptome profile of *S. japanicum.* The results obtained here will help to effects of alternate prey (*C. cephalonica* eggs) on development and reproduction success of *S. japanicum.*

## Results

### Comparison of biology and life history parameters of *S. japonicum* feeding on different hosts

The development periods of 2nd, 3rd, 4th larval instars, and pupa as well as 2nd instar to adult emergence of *S. japonicum* feeding on *C. cephalonica* eggs were significantly longer as compared to those feeding on immature whitefly (Table 1). The *C. cephalonica* eggs had significantly lower survival rate during the second instar as compared to those feeding on immature whitefly (*p* < 0.05 by t-test).

**Table 1:** Life history parameters (± S.E) of *S. japonicum* feeding on *B. tabaci* and *C. cephalonica* eggs

Life history traits	Diet treatments			
	*B. tabaci*	*C. cephalonica* eggs		*P* value
Development time of second instar (days)	1.51(± 0.05)		2.31(± 0.04)	0.000
Development time of third instar (days)	1.86(± 0.10)		2.55(± 0.08)	0.006
Development time of fourth instar (days)	3.85(± 0.06)		4.92(± 0.11)	0.001
Development time of pupa (days)	4.12(± 0.05)		4.89(± 0.03)	0.000
Development time of second instar to adults (days)	15.14(± 0.14)		19.48(± 0.11)	0.000
Survival rate of First instar (%)	97.78(± 1.11)		23.12 (± 0.29)	0.001
Survival rate of second instar (%)	93.18(± 0.08)		78.89(± 4.84)	0.042
Survival rate of third instar (%)	96.3(± 2.14)		88.92(± 2.18)	0.073
Survival rate of fourth instar (%)	98.81(± 1.19)		95.22(± 2.64)	0.282
Survival rate of pupa (%)	98.77(± 1.23)		98.15(± 1.85)	0.795

**Fig. 1:**
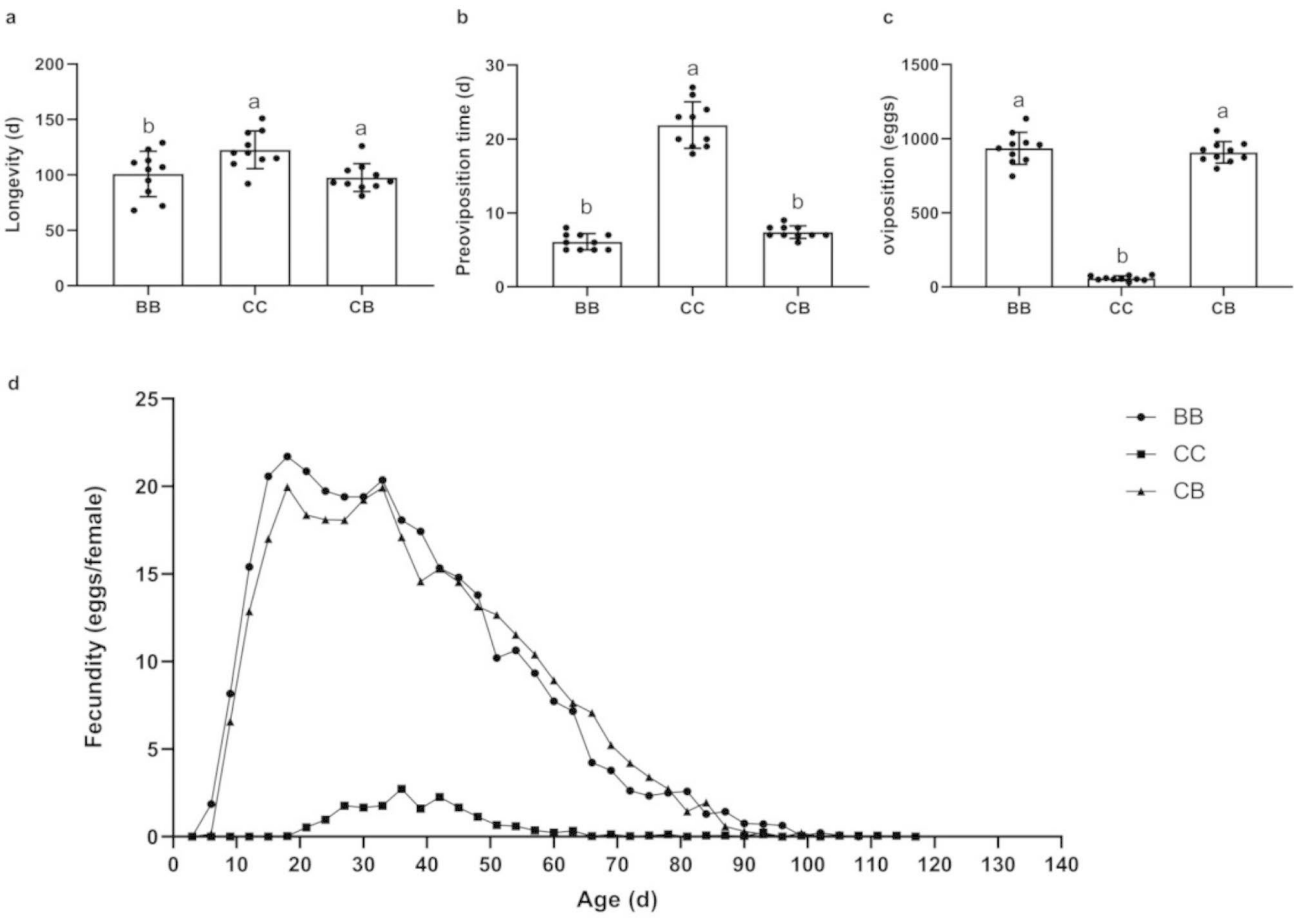
Longevity and reproductive capacity of *S. japonicum* adult fed with different prey. BB: *S. japanicum* fed with *B. tabaci* at both larval and adult stages; CC: *S. japanicum* fed with *C. cephalonica* eggs at both larval and adult stages; CB: *S. japanicum* fed with *C. cephalonica* eggs in the larval stage and *B. tabaci* in the adult stage. (a): Longevity. (b): Pre-oviposition time. (c): oviposition. (d): Average daily oviposition. The data were analyzed with one-way analysis of variance. The bars show mean ± SD, different letters over the bars mean significant differences (P < 0.05)

The longevity of *S. japonicum* feeding on *C. cephalonica* eggs was significantly higher from those feeding on immature whitefly (Fig. 1A). The pre-oviposition period of *S. japonicum* feeding on *C. cephalonica* eggs (21.90 ± 0.99 d) was significantly higher than those feeding on immature whitefly (6.10 ± 0.35 d). The pre-oviposition period of *S. japonicum* feeding on *C. cephalonica* eggs was significantly similar to those feeding on *C. cephalonica* eggs during the larval stage and *B. tabaci* in the adult stage (Fig. 1B, C).

**Fig. 2:**
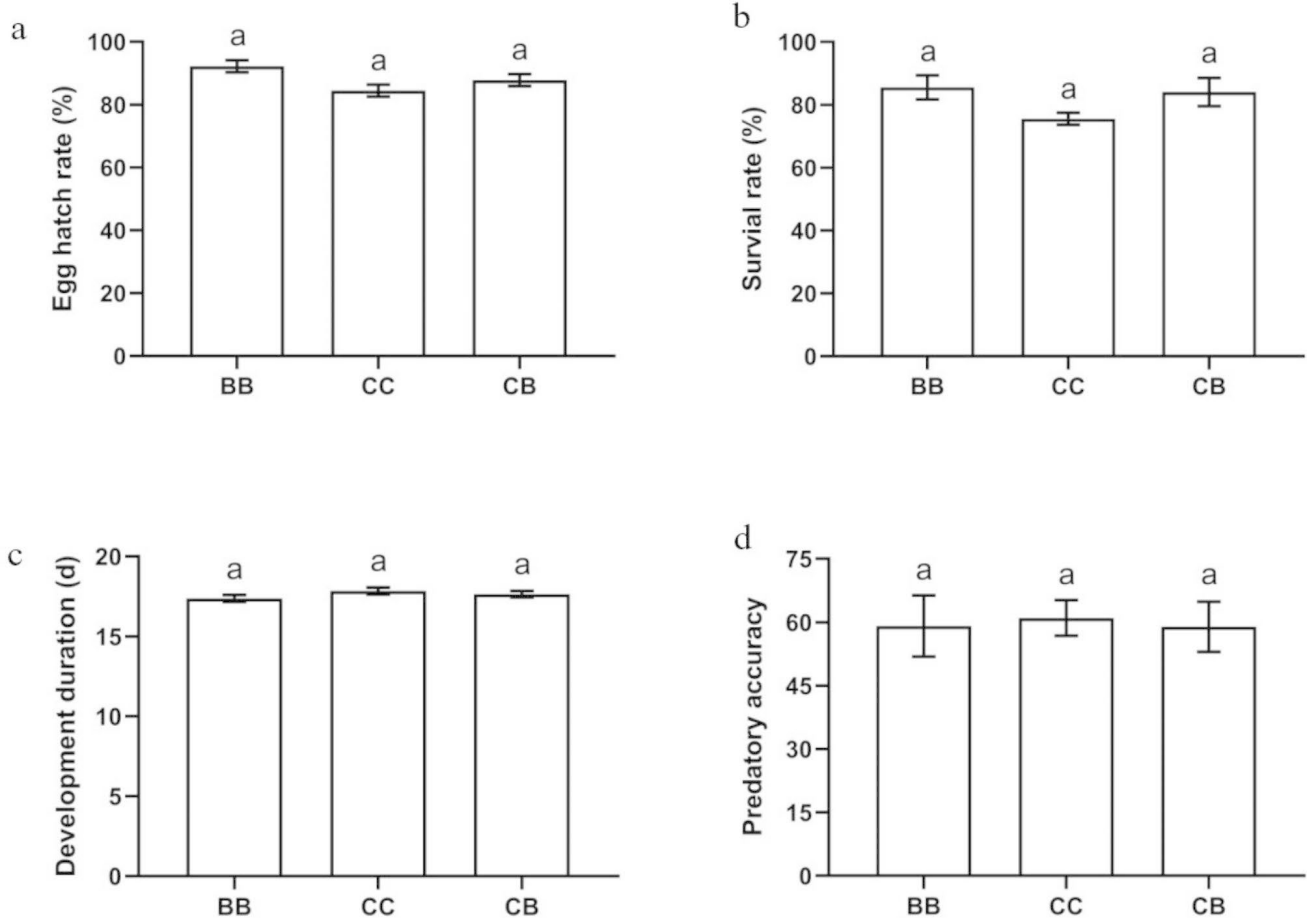
Growth and predation of first generation of *S. japanicum*. (a): Egg hatch rate. (b): Survival rate. (c): Development duration. (d): Predatory accuracy. The data were analyzed with one-way analysis of variance. The bars show mean ± SD, different letters over the bars mean significant differences (P < 0.05)

The oviposition period of *S. japonicum* adults feeding on *C. cephalonica* eggs (68.33 ± 1.97 d) was significantly shorter than those feeding on immature whitefly (76.00 ± 2.45 d) whereas oviposition period of *S. japonicum* feeding on *C. cephalonica* eggs during the larval stage and *B. tabaci* in the adult stage was significantly similar to those feeding on *B. tabaci* (Fig. 1C). Similarly, the fecundity of *S. japonicum* adults feeding on *C. cephalonica* eggs was significantly lower than those feeding on immature whitefly while on contrary the fecundity of *S. japonicum* feeding on *C. cephalonica* eggs during the larval stage and *B. tabaci* in the adult stage was significantly similar to those feeding on *B. tabaci* (Fig. 1D). The hatching rate, survival and developmental period of eggs laid by *S. japonicum* adults feeding on *C. cephalonica* eggs were significantly similar with the hatching rate of eggs deposited by immature whitefly fed females (Fig. 2A, B, & C). The predatory efficacy of 4th instar larvae from F1 generation of *C. cephalonica* eggs fed *S. japonicum* was significantly similar to the 4th instar larvae from F1 generation of whitefly fed *S. japonicum* (Fig. 2D).

### Summary of RNA-seq datasets

Raw sequences were obtained for six samples across two groups, including three biological replicates per group (Additional file 1 A). The SeqPrep and Sickle softwares were used to remove linker sequences, low-quality reads, sequences with high proportions of “N” nucleotides (representing uncertain base information), and excessively short sequences. In total, we obtained high-quality clean data with base quality values Q20 and Q30 greater than 98.95% and 96.30%, respectively. The GC content was > 40%, and the sequencing error rates were < 0.03% (Additional file 1B). According to the length distribution of unigenes, the largest gene length was 200–500 bp (15,286 unigenes; 51%), the second largest gene length was 501–1000 bp (5564 unigenes; 18%), and the smallest gene length was 4000–4500 bp (286 unigenes; 1%) (Additional file 1 C). The correlation coefficients (Persons’ correlation) showed highly reporducible data across different replications (Additional file 1D).

**Fig. 3:**
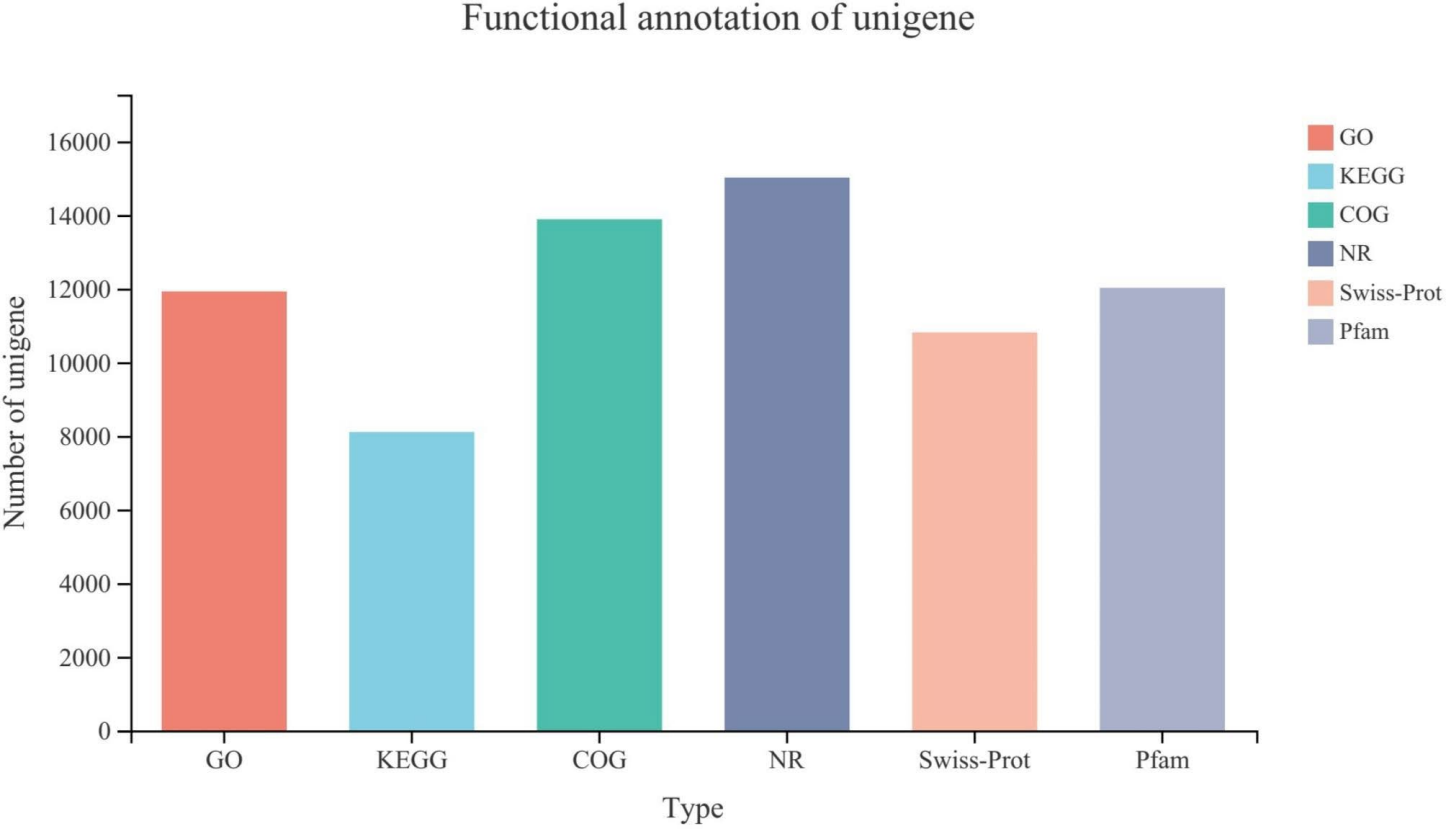
Gene annotation of each database

The unigenes were compared with six databases (NR, Swiss prot, Pfam, eggnog, go and KEGG), and the annotation information of gene/transcript was obtained. A total of (15,837, 52.50%) genes were annotated in this experiment, with GO database matching (12,038, 39.91%), followed by KEGG (8176, 27.11%), COG (14,026, 46.50%), NR (15,193, 50.37%), Swiss-Prot (10,887, 36%) and PFAM (12,106, 40.13%) database (Fig. 3, Additional file 1E).

### 3.5 Analysis of DEGs

**Fig. 4:**
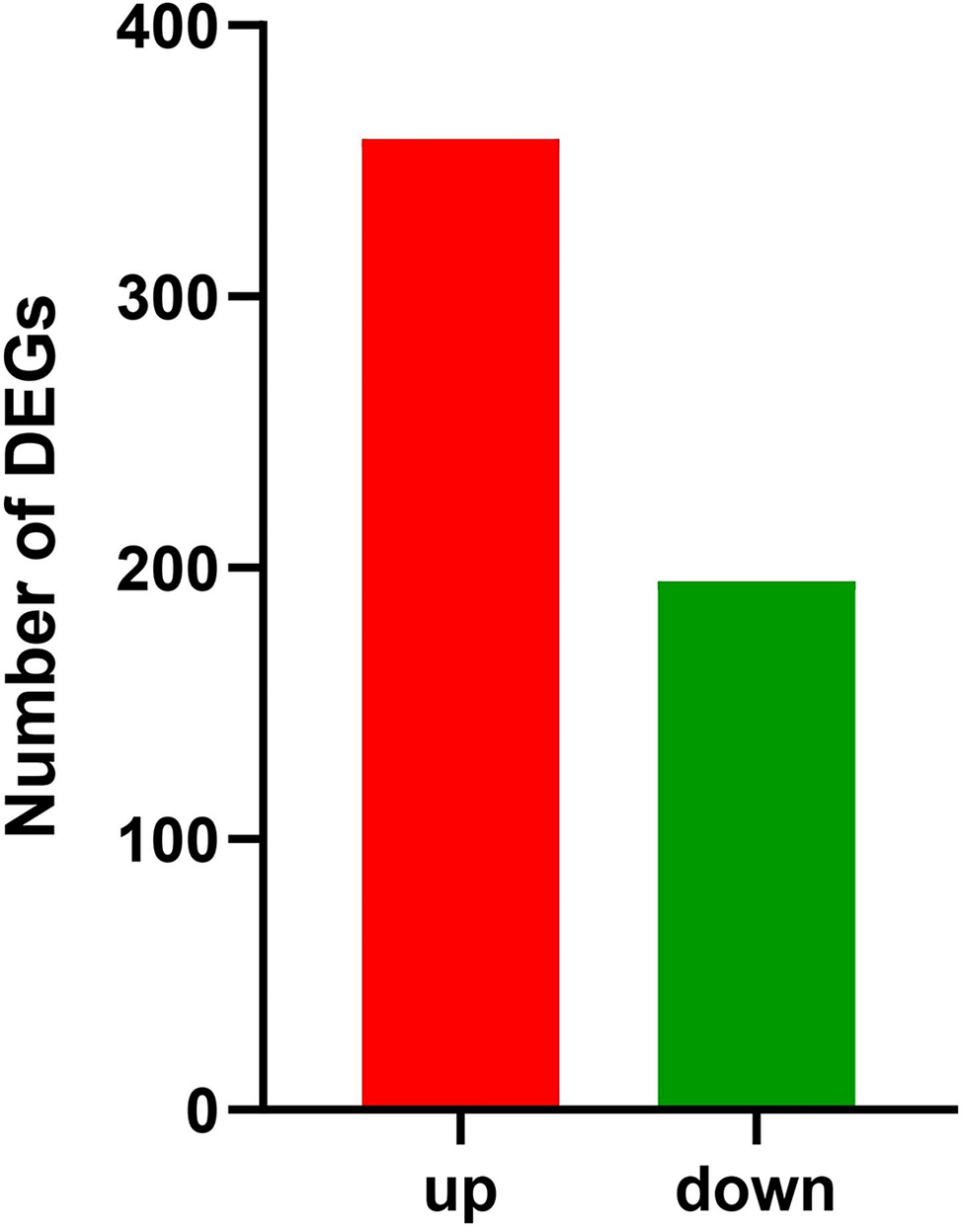
Summary of differently expressed genes (DGEs) in *C. cephalonica* eggs fed *S. japonicum*

The differentially expressed genes of *S. japonicum* feeding on *C. cephalonica* eggs compared with whitefly fed *S. japonicum* were identified by using the number of fragments per kb per million (FPKM) of clean reads. Relative to control genes with (FDR) ≤ 0.001 and |log2FC| >=1.000 were recognized as differentially expressed genes. Our result showed differential expression of 553 (358 up-regulated and 195 down-regulated) DEGs in *S. japonicum* feeding on *C. cephalonica* eggs compared with whitefly fed *S. japonicum* (Fig. 4), among which the genes with in the Log2FC values in the range of 1.0 to 2.0 were in majority (236 DEGs), followed by 2.0-9.41 (122 DEGs), <-1.0 and >-0.3 (11 DEGs) and the number of genes with Log2FC values <-3.0 were 84 (2.81%) (Additional file 2 A).The cluster analysis differentially expressed genes showed that the log2FC values of most genes in treatment groups were positive, which indicated that these genes were up-regulated compared with those in control groups (Additional file 2B).

A vast majority of up-regulated genes (358) were observed in *S. japonicum* feeding on *C. cephalonica* eggs compared with whitefly fed *S. japonicum*. Among the 358 up-regulated DEGs, the major identified categories included: 114 orphan genes (unknown proteins), 51 uncharacterized proteins, 17 hypothetical proteins, 8 cytochrome P450 genes, 4 odorant binding proteins and 3 apolipophorin (Additional file 2B). The major up-regulated DEGs expressed in *S. japonicum* feeding on *C. cephalonica* eggs compared with whitefly fed *S. japonicum* were sterol O-acyltransferase 1-like, apolipophorin, partial, and zinc metalloproteinase nas-13 having Log2FC value in range of 4–6 (Table 2).

**Table 2:** Top differentially expressed genes (DEGs) in *C. cephalonica* eggs fed *S. japonicum* adults compared with whitefly fed *S. japonicum adults*

Gene ID	Annotation	Whitefly fed *S. japonicum*	*C. cephalonica* eggs fed *S. japonicum*	FC	Log_2_FC	P-adjust
TRINITY_DN16291_c0_g1	sterol O-acyltransferase 1-like	0.02	1.4	72.5	6.1	8.18E-05
TRINITY_DN6870_c0_g2	unknown protein	0.1	3.8	30.5	4.9	0.004031801
TRINITY_DN27101_c0_g1	Membrane-bound alkaline phosphatase-like	2.0	61.4	29.9	4.9	7.07E-27
TRINITY_DN29302_c0_g1	unknown protein	0.1	3.8	29.3	4.8	0.01236846
TRINITY_DN8511_c0_g2	unknown protein	0.07	2.1	29.3	4.8	0.00531795
TRINITY_DN9895_c0_g1	unknown protein	0.04	2.0	29.2	4.8	0.004899387
TRINITY_DN1001_c0_g2	unknown protein	0.1	3.0	26.0	4.7	0.006097372
TRINITY_DN20712_c0_g1	unknown protein	0.1	2.7	25.7	4.6	0.018393664
TRINITY_DN23644_c0_g1	apolipophorin, partial	0.06	1.5	23.3	4.5	1.75E-06
TRINITY_DN4963_c0_g1	zinc metalloproteinase nas-13	1.3	21.9	17.2	4.1	2.01E-09
TRINITY_DN8580_c0_g1	unknown protein	21.3	0.3	0.01	-5.9	0.045951459
TRINITY_DN22634_c0_g1	unknown protein	4.2	0.06	0.01	-5.9	4.50E-05
TRINITY_DN5806_c0_g1	unknown protein	3.5	0.08	0.02	-5.1	0.001686944
TRINITY_DN11511_c0_g1	unknown protein	6.4	0.2	0.03	-4.9	0.003699523
TRINITY_DN2450_c0_g2	unknown protein	3.0	0.1	0.03	-4.7	0.000329698
TRINITY_DN29420_c0_g1	unknown protein	4.8	0.2	0.03	-4.7	0.017722915
TRINITY_DN18359_c0_g1	unknown protein	16.2	0.8	0.04	-4.5	0.025205911
TRINITY_DN21887_c0_g2	beta isoform isoform X46	1.2	0.05	0.04	-4.4	0.01589911
TRINITY_DN15784_c0_g2	titin isoform X1	1.6	0.07	0.05	-4.3	0.024289713
TRINITY_DN29287_c0_g1	Transposable element P transposase	1.0	0.06	0.06	-4.0	0.017937259

Among the 195 genes that were down-regulated in *S. japonicum* feeding on *C. cephalonica* eggs compared with whitefly fed *S. japonicum*, 124 were classified as orphan genes. Further major categories of down-regulated DEGs in *S. japonicum* feeding on *C. cephalonica* eggs consisted of 24 uncharcterized proteins, 10 hypothetical proteins and 2 cuticle proteins (TRINITY_DN9112_c0_g1; TRINITY_DN8579_c0_g1). The major down-regulated DEGs expressed in *S. japonicum* feeding on *C. cephalonica* eggs compared with whitefly fed *S. japonicum* were beta isoform isoform X46, titin isoform X1, and Transposable element P transposase (Table 2).

### GO annotation analysis of DEGs

**Fig. 5:**
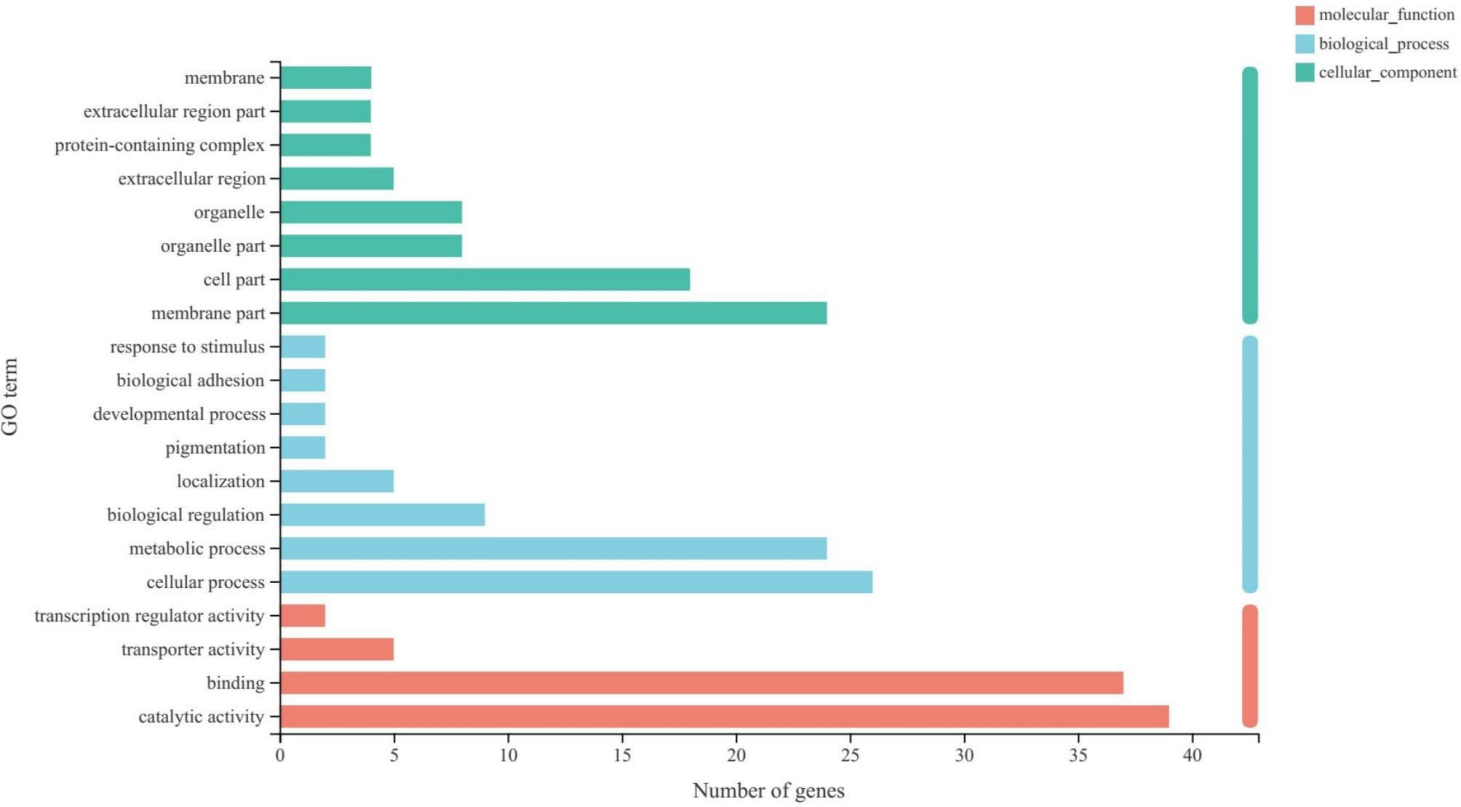
Gene ontolgy (GO) enrichment of differentially expressed genes in *C. cephalonica* eggs fed *S. japonicum*

To further examine the functions of the DEGs, we annotated the genes using the GO database into the three major categories: biological process, cellular component, and molecular function (Fig. 5). GO annotation analysis showed that the three most representative GO categories in cellular components were “membrane part”, “cell part”, and “organelle part”. In the biological process term, several DEGs were involved in the “cellular process (26 DEGs)”, and “metabolic process (24 DEGs)”. In the molecular function term, DEGs mainly participated in “catalytic activity” and “binding” (Fig. 5).

### KEGG pathway analysis

**Fig. 6:**
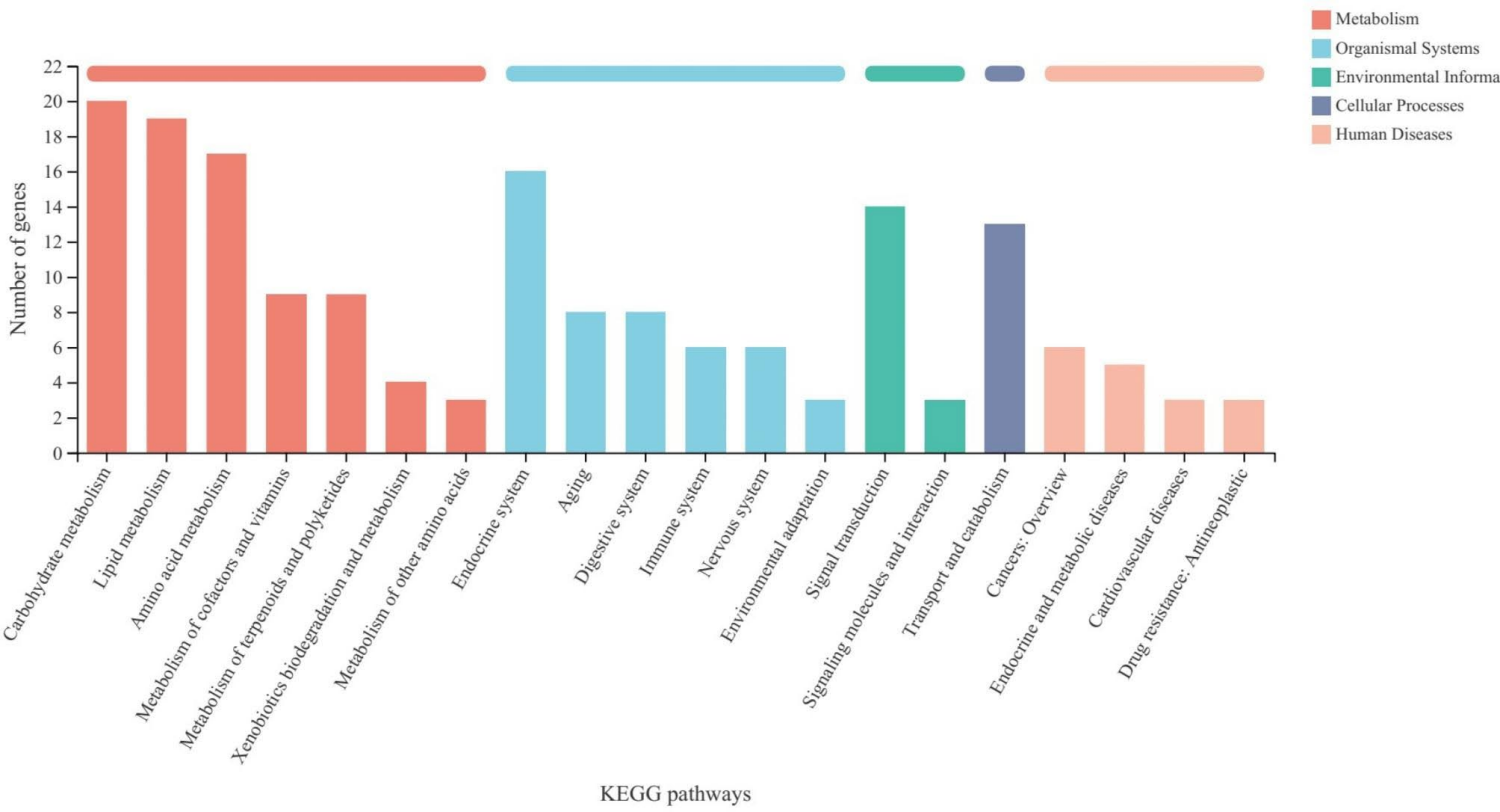
Summary of KEGG reference pathways of *C. cephalonica* eggs fed *S. japonicum*

The KEGG (Kyoto Encyclopedia of Genes and Genomes) pathway enrichment analysis of DEGs was performed to identify the potential pathways that were up- and down-regulated in *S. japonicum* feeding on *C. cephalonica* eggs compared with whitefly fed *S. japonicum*. Totally, 90.78% (325 out of 358) of total number of up-regulated DEGs in *S.japonicum* feeding on *C. cephalonica* eggs were annotated to KEGG pathways. Three major categories of pathways most represented as up-regulated in *S. japonicum* feeding on *C. cephalonica* eggs were (1) metabolism, (2) organismal systems, and (3) Human diseases (Fig. 6 & Additional file 4). The categories of DEGs showing differences in the number of genes among different treatments or those having the most differential expression are highlighted herein.

### Metabolism

Major differences were observed within the metabolism category between *S. japonicum* feeding on *C. cephalonica* eggs compared with whitefly fed *S. japonicum*. In total, 133 DEGs belonging to 58 metabolic pathways (all up-regulated) were found to be differentially expressed in *S. japonicum* feeding on *C. cephalonica* eggs (Additional file 4). The majority of DEGs were expressed in metabolic pathways related to carbohydrate metabolism, lipid metabolism, and amino acid metabolism (Fig. 6 & Additional file 4).

### Organismal systems

In total, 72 DEGs belonging to 41 organismal systems pathways (all up-regulated) were found to be differentially expressed in *S. japonicum* feeding on *C. cephalonica* eggs (Additional file 4). The majority of DEGs were expressed in organismal systems pathways related to endocrine system, aging, digestive system, immune system and nervous system (Fig. 6 & Additional file 4).

### Signal transduction

Among genes annotated to signal transduction pathways, 29 up-regulated DEGs belonging to 13 pathways were observed in *S. japonicum* feeding on *C. cephalonica* eggs (Additional file 4). The majority of DEGs were expressed in AMPK signalling pathway, PI3K-Akt signalling pathway, Ras signalling pathway, and MAPK signalling pathway (Additional file 4).

### Transport and catabolism

Among genes annotated to transport and catabolism pathways, 13 up-regulated DEGs were observed in belonging to 5 pathways were observed in *S. japonicum* feeding on *C. cephalonica* eggs (Additional file 4). The DEGs were expressed in lysosome, peroxisome, phagosome, and endocytosis pathways (Additional file 4).

### Quantitative reverse transcription PCR validation of DEGs

**Fig. 7:**
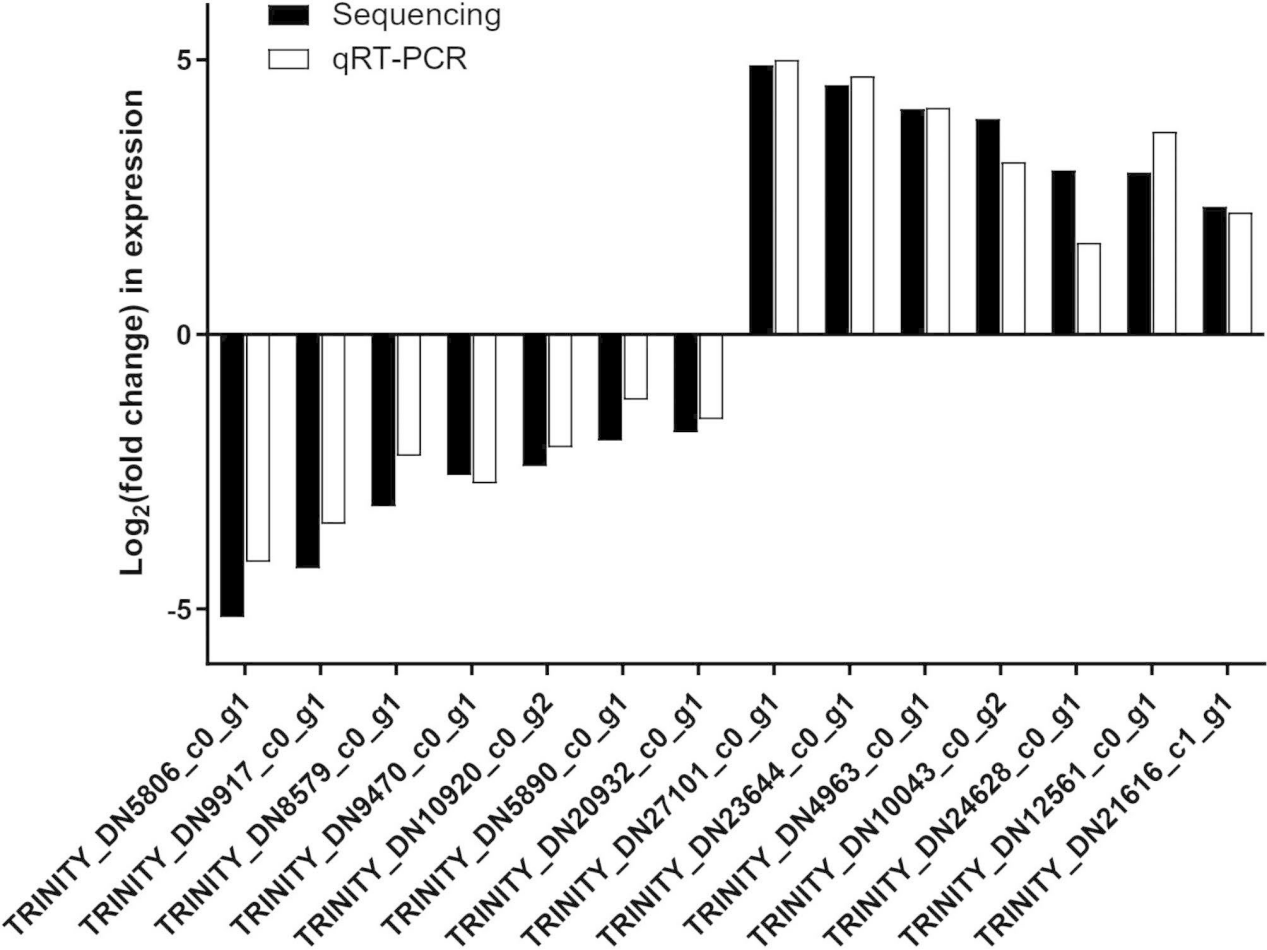
qRT-PCR validation of DEGs expressed in *C. cephalonica* eggs fed *S. japonicum*. Error bars: SD of the mean of three biological replicates

The accuracy of DEGs observed through RNA sequencing was verified by Quantitative RT-PCR (qRT-PCR) analysis. In total 15 genes were randomly selected and the results showed similar expression patterns to DEG analysis (Fig. 7).

## Discussion

The mass production of natural predators with prolonged shelf life is a prerequisite for their field application as pest control agents [1, 2]. The traditional methods used for the mass production of insect predators rely heavily on the consistent supply of natural prey and any fluctuation in natural prey supply can challenge the maintenance of the complex three-level nutrient system for the mass production of insect predators [4]. Thus, developing methods of mass production of insect predators using cheaper food sources can help promote the application of these biological pest control agents. *Serangium japonicum* (Coleoptera; Coccinellidae) is an obligate predator of multiple whitefly species [15]. The mass production of *S. japonicum* rely heavily on the consistent supply *B. tabaci*, the screening/search of alternate food sources for mass production of *S. japonicum* can be an area of high interest to promote the field application of *S. japonicum. Corcyra cephalonica* is a globally distributed stored grain pest and its eggs are widely used for the mass production of different biological control agents. This study explains the effects of *B. tabaci* (natural prey) and *C. cephalonica* eggs (alternative food) on life history and transcriptome profile of *S. japanicum.*

This study revealed that *S. japonicum* was able to successfully complete its life cycle while feeding on *B. tabaci* (natural prey) and *C. cephalonica* eggs (alternative food), although *S. japonicum* has long been considered as the specialist predator of *B. tabaci.* In this study, *C. cephalonica* eggs fed *S. japonicum* individuals had longer developmental period as compared to those feeding on whitefly but the survival rates (3rd instar nymphs, 4th instar nymphs and pupae) of *C. cephalonica* eggs fed *S. japonicum* individuals were significantly similar to those feeding on whitefly. The life history attributes of *S. japonicum* adults (fecundity, preoviposition periods etc.) feeding on *C. cephalonica* eggs was significantly lower than those feeding on immature whitefly while on contrary these parameters of *S. japonicum* feeding on *C. cephalonica* eggs during the larval stage and *B. tabaci* in the adult stage was significantly similar to those feeding on *B. tabaci.* The predatory efficacy of *C. cephalonica* eggs fed *S. japonicum* individuals were significantly similar to other treatments. These changes in in life history can be attributed to differences in chemical composition, body size, and mobility of the prey [22]. These changes in life history traits also induce differential expression of development related genes during adaptation to diet shits [23].

Based on the prey type classification proposed by Hodek and Honĕk [24], both of our tested prey would be classified as ‘acceptable’ for *S. japonicum*. The two prey species were readily consumed by larvae and adults of *S. japonicum*, regardless of the physiological consequences. However, *C. cephalonica* eggs would be considered a marginal prey [25]. Although, such marginal prey does not directly support reproduction, they may sustain predators when essential prey is in short supply [26, 27]. When feeding on an ‘alternative’ prey, a predator may cease oviposition until conditions improve and essential prey compose enough of the diet to support reproduction [28]. The above-mentioned changes in survival rates supports the possibility of using *C. cephalonica* eggs for mass production of *S. japonicum* during periods of whitefly shortage.

As discussed above the life history characteristics differed between *C. cephalonica* eggs-fed and whitefly fed *S. japonicum* although the rates of prey consumption by 4th instar F1 pro-genies of *C. cephalonica* eggs-fed and whitefly fed *S. japonicum* were significantly similar to each other. The transcriptome analysis revealed differential expression of several genes related to growth and reproduction. For reduced fecundity, two haemolymph juvenile hormone binding proteins (protein takeout-like, TRINITY_DN25254_c0_g1; PREDICTED: protein takeout-like; TRINITY_DN25254_c0_g1) were down-regulated in *C. cephalonica* eggs-fed females of *S. japonicum* marking the deficiency of juvenile hormone in diet. It may be that the amount of was insufficient. Juvenile hormones affects the development of female reproductive system and vitellogenesis in a variety of predatory ladybird [29–32]. In addition, vitellogenesis in insects is regulated by endocrine hormones, and the nutrition plays an important role in most female insects [33]. The absence of one or more nutrients can affect the transcriptional synthesis of vitellogenin. Our results up-regulation of one vitellogenin gene (*vitellogenin 2, TRINITY_DN3221_c0_g1*) in *C. cephalonica* eggs-fed females of *S. japonicum* compared to whitefly fed *S. japonicum* females, indicating the suitability of nutrition in the *C. cephalonica* eggs for vitellogenesis.

In response to dietary changes, *S. japanicum* may successfully feed on *C. cephalonica* eggs by removing or transforming toxic chemicals. As a result, detoxification-related genes such as cytochrome P450 can play an important role in this process. Cytochrome P450 is the most important functional component of multifunctional oxidase [34]. It plays a key role in the detoxification of exogenous substances, catabolism of xenobiotics, cell metabolism, and homeostasis, and is an extremely important component of the metabolic system [35, 36]. Our results showed upregulation of 8 cytochrome P450s (cytochrome P450 9Z4, TRINITY_DN4163_c0_g3; cytochrome P450 345B1,TRINITY_DN17914_c0_g1; PREDICTED: cytochrome P450 9e2-like,TRINITY_DN8154_c0_g1; cytochrome P450 9e2-like, TRINITY_DN4163_c0_g1; cytochrome P450 6d4 isoform X2,TRINITY_DN7689_c0_g1; cytochrome P450 6k1, partial,TRINITY_DN10461_c0_g1; cytochrome P450 family 4 subfamily Q polypeptide 4,TRINITY_DN836_c0_g1; cytochrome P450 9e2-like, TRINITY_DN4163_c0_g2) in *S. japonicum* feeding on *C. cephalonica* eggswhich might be related to the prolonged larvae development time [36]. Furthermore, three GOBP family genes (*odorant binding protein 22, TRINITY_DN4732_c0_g1; odorant binding protein C20, TRINITY_DN19721_c0_g1; general odorant-binding protein 70 isoform X2, TRINITY_DN3783_c0_g1*) were upregulated which might have led to foraging behavior, alertness, and feeding of *C. cephalonica* eggs-fed females of *S. japonicum* significantly similar to whitefly fed *S. japonicum* [37].

Furthermore, our results revealed the up-regulation of DEGs related to development, nutrition, storage and transport. Up-regulation of three Apolipophorin DEGs was observed in *C. cephalonica* eggs-fed females of *S. japonicum* which are known to act as vehicles of lipid transport in different insect species [38]. Hexamerins are the insect proteins involved in metamorphosis and other related functions [39–41]. Our results revealed up-regulation of hexamerin 4 precursor (TRINITY_DN2562_c0_g1). Phosphoenolpyruvate carboxykinase protein (PECPK, annotated as TRINITY_DN9105_c0_g2) is a catalyst involved in lactate gluconeogenesis [38]. Our results revealed upregulated expression of PEPCK. PEPCK is involved in indirect enhancement of insect glucose levels. may indirectly enhance glucose levels and provide nutrients to adult insects Specifically, PEPCK converts oxaloacetate into phosphoenolpyruvate and carbon dioxide [42].

The transcriptome results showed that the genes enriched in carbohydrate metabolism, lipid metabolism, and amino acid metabolism pathways were significantly upregulated. The balance of nutrients in the diet has a significant impact on the growth and reproduction of insects [43, 44]. Our results suggested that *S. japanicum* may lack key nutrients when feeding on *C. cephalonica* eggs, which results in corresponding changes in the genetic regulation of nutrient transport and metabolism.

We found that most genes enriched in lysosome, peroxisome, phagosome, and autophagy pathways were upregulated, which may be related to the nutrition provided by the *C. cephalonica* eggs. Nutritional status is known to affect oviposition in several insects [45]. Vitellogenin biosynthesis and the process of oviposition are associated with the nutritional status of the insects [46, 47]. When nutrients are insufficient, cells trigger autophagy to degrade relatively redundant proteins and organelles in order to provide materials and energy for survival. Autophagy is a catabolic process that requires the use of lysosomes to degrade excess cellular components and organelles [48]. The co-activation of autophagy and lysosomes is regulated by a variety of transcription factors, such that an increase in autophagy enhances the biosynthesis and functioning of lysosomes [49]. Simultaneously, nutritional stress can also induce apoptosis. In normal conditions, apoptosis is induced in the later stages of oogenesis when nurse cells degenerate after completing their functions [50]. However, nutritional deficiency can induce apoptosis in the egg chamber at the eighth and ninth stages, and the available nutrients are used to cultivate fewer normal eggs in *Drosophila* [51].

## Conclusion

This study revealed that *S. japonicum* was able to successfully complete its life cycle while feeding on *B. tabaci* (natural prey) and *C. cephalonica* eggs (alternative food). The *C. cephalonica* eggs fed *S. japonicum* individuals had longer developmental period and lower fecundity as compared to those feeding on whitefly but the survival rates (3rd instar nymphs, 4th instar nymphs and pupae) and predatory efficacy of *C. cephalonica* eggs fed *S. japonicum* individuals were significantly similar to to those feeding on whitefly. Transcriptome analysis showed that when faced with dietary changes, *S. japanicum* could successfully feed on *C. cephalonica* eggs by regulating genes related to nutrient transport, metabolism, and detoxification. Moreover, *S. japanicum* degraded excess cellular components through ribosomal autophagy and apoptosis, which provided sufficient materials and energy for survival and basic metabolism. These findings are of great significance for studying the functional evolution of *S. japanicum* in response to dietary changes.

## Methods

### Plants and insects

Seeds of cotton *Gossypium hirsutum* L. (Malvales: Malvaceae) Luman No. 32 were obtained from Cotton Research Centre, Shandong Academy of Agricultural Sciences, Jinan, China. Healthy seedlings were cultivated within plastic pots (20 cm diameter) in screened cages and grown to approximately 30 cm in height before being used in experiments.

The *B. tabaci* biotype B (MEAM) populations used in this study were reared on cotton plants following Wang et al. [52]. The insects were reared for several generations under laboratory conditions at the Provincial Key Laboratory of Biopesticides and Innovation, South China Agricultural University, Guangzhou, P. R. China.

Adult females of *S. japonicum* were released into cages containing cotton seedlings bearing *B. tabaci.* After oviposition, the cotton leaves (containing *S. japonicum* eggs and *B. tabaci*) were transferred to Petri dishes (diameter = 60 mm; height = 10 mm), which were placed in an artificial climate chamber (26 ± 2 ℃, 70 ± 5% relative humidity [RH], 14 L:10D photoperiod). The newly hatched *S. japonicum* nymphs were fed on *B. tabaci* nymphs until emergence as adults.

*Corcyra cephalonica* eggs purchased from Guangzhou Yuefeng Biological Control Technology Co., Ltd. were reared for several generations by following an improved methodology of Bernardi et al. [53]. The larvae were reared in the 455 mm × 325 mm × 40 mm box with the proportion of corn meal: soybean meal: wheat bran at 7:2:1 by weight indoors (26 ± 2 ℃, 60–80% relative humidity [RH]). After the larvae emerged, the adults were collected in a 100 mm×50 mm gauze bag to lay eggs. The *C. cephalonica* eggs were collected daily and inactivated with a UV lamp before use in experiments.

### Comparison of biology and life history parameters of *S. japonicum* feeding on different hosts

*Serangium japonicum* adults (10 pairs) from the laboratory culture were placed on cotton leaves bearing whitefly eggs for oviposition. Leaves with beetle eggs (< 12 h old) were excised from the main plants and placed in 6 cm diameter plastic Petri dishes lined with moistened filter papers (8 cm in diameter) at the bottom of the dish. Cohorts of at least 60 eggs each were assigned to two groups with three replicates on fresh leaf disks of cotton (10-15cm^2^) bearing immature whitefly; eggs were monitored daily until the emergence of 2nd instar nymphs. Neonates were gently removed from the leaves and transferred using a fine hairbrush (No.00) to fresh leaf disks of cotton leaf of 10-15cm^2^ bearing immature whitefly or *C. cephalonica* eggs in a Petri dish. The Petri dishes were then placed in growth chambers (PXY-300QA, Shaoguan Keli Experimental Instrument Co., Ltd., Shaoguan, Guangdong) at 26 ± 1 ºC, at a relative humidity of 75 ± 10% and a photoperiod of 14:10 (L: D). Leaf disks were replaced daily except during the pupal stage. Beetles were monitored daily for molting of the different developmental stages and mortality occurring at each stage, until adult emergence.

Newly emerged adults were individually placed on a leaf disk having different food. Adults fed on *B. tabaci* in the larval stage continued to be fed on *B. tabaci*, and adults fed on *C. cephalonica* eggs in the larval stage were divided into two groups and fed on *B. tabaci* and *C. cephalonica* eggs, respectively. The three groups were named BB, CB and CC. Through observation, a total of 10 randomly selected pairs with mating behavior were placed separately on a leaf disk of 5-8 cm diameter with a moistened filter paper lined at the bottom of a Petri dish and kept at 26 ± 1 ºC in the growth chamber. Fresh leaf disks with whitefly immatures or *C. cephalonica* eggs were provided daily as a source of food. The number of eggs laid, the number of adults surviving each day and the longevity of the adults were recorded daily until all beetles died. The sex of each adult was determined after death by dissecting and exposing the reproductive organs.

The effects of different feeding hosts on development and predatory efficacy of F1 *S. japonicum* were also observed. The eggs laid by *S. japanicum* females from different treatment groups were transferred to plastic Petri dishes containing *B. tabaci*-bearing cotton leaves using a camel hair brush. To maintain the relative humidity and aeration, the cotton leaves were placed on moistened filter paper and the Petri dishes were covered with a plastic lid with small holes. The experimental setup was incubated at 26 ± 2 ℃ and 70 ± 5% R.H. under a 14 L:10D photoperiod. The leaves bearing *B. tabaci* nymphs were changed daily to ensure an ample supply of food. Beetles were monitored daily for growth and development until adult emergence. Thirty eggs of S. japonicum were monitored per treatment group, and three replicates were established. In addition, the 4th instar *S. japanicum* larvae from each treatment group were individually placed in a Petri dish. After 12 h of starvation, the larvae were transferred to a new Petri dish containing cotton leaves bearing 4th instar *B. tabaci* nymphs (100 individuals). The *S. japanicum* larvae were allowed to feed for 24 h, and we counted the number of 4th instar *B. tabaci* nymphs consumed by each larva. Each treatment contained 10 *S. japanicum* larvae.

### Comparison of *S. japonicum* transcriptome feeding on different hosts by RNA-sequencing

The freshly emerged 1st instar nymphs (from one group each) were assigned to two feeding treatments/hosts; immature whitefly and *C. cephalonica* eggs on fresh leaf disks of cotton (10-15cm^2^) placed in 9 cm diameter plastic Petri dishes lined with moistened filter papers (8 cm in diameter) at the bottom of the dish. The Petri dishes were then placed in growth chambers (PXY-300QA, Shaoguan Keli Experimental Instrument Co., Ltd., Shaoguan, Guangdong) at 26 ± 1 ºC, at a relative humidity of 75 ± 10% and a photoperiod of 14:10 (L: D). Leaf disks were replaced daily (except during the pupal stage) until adult emergence. The freshly emerged adults from each treatment were collected in 1.5 mL Eppendorf tubes, frozen in liquid nitrogen, and stored at -80 °C. Three biological replicates were performed for each group, and each replicate contained 5 adults. RNA extraction and sequencing was performed by Shanghai Major Biomedical Technology Co., Ltd. Total RNA was extracted from the sample, and a Nanodrop 2000 spectrophotometer was used to detect the concentration and purity of the extracted RNA. Agarose gel electrophoresis was used to detect the integrity of the RNA, and the Agilent2100 system was used to determine the RIN value. After total RNA extraction, the mRNA was separated using magnetic oligo (dT) beads and fragmented randomly using fragmentation buffer. Small fragments (approximately 300 bp in length) were screened with magnetic beads and reverse transcribed into cDNA. End-Repair Mix was used for terminal repair and joint PCR amplification. The cDNA library was created by PCR amplification and sequenced on an Illumina platform.

### Enrichment analysis of unigenes and DEGs

The raw sequences were analyzed using the DESeq2 software by specifying a negative binomial distribution and the following parameters: *p*-adjust < 0.05 and |log_2_FC|≥1. The *p*-adjust was calculated as the *p*-value after Benjamini and Hochberg correction for multiple tests. These analyses were used to identify DEGs between the treatment and control groups.

Both gene ontology (GO) enrichment analysis and the Kyoto Encyclopedia of Genes and Genomes (KEGG) pathway enrichment analysis [54] passed Fisher’s exact test, and *P*-values were corrected by a Bonferroni correction for multiple tests. When the corrected *P*-value (corrected for false discovery rate) was < 0.05, the GO function or KEGG pathway was considered significantly enriched.

### Quantitative real-time PCR validation

DEGs screened by transcriptome sequencing were randomly selected for quantitative real-time PCR (qRT-PCR) validation (Primer details are available in Table 3). Three biological and three technical replicates were performed. RNA was extracted from *S. japanocum* using TRIzol reagent according to the manufacturer’s directions. The cDNAs were prepared using 1 µg of RNA from various samples with the PrimeScriptTM RT reagent Kit with gDNA Eraser (RR047A, Takara, Japan). Then, the cDNAs were diluted ten-fold before the RT-qPCR reactions. The RT-qPCR primers were designed and synthesized by Sangon Biotech (Shanghai) Co., Ltd., and are listed in Table 3. The qRT-PCR mixture (10 µL) consisted of SYBR Green qPCR Mix (5 µL), cDNA (2 µL), upstream and downstream primers (0.5 µL each), and RNase-free water (2 µL). The qRT-PCR reaction conditions were as follows: pre-denaturation at 95 ℃ for 3 min, and 39 cycles of denaturation at 95 ℃ for 10 s, annealing at 60 ℃ for 20 s, and extension at 72 ℃ for 30 s. The 2^-ΔΔCt^ technique was used to calculate relative expression levels using β-actin as an internal control [55, 56].

**Table 3:** Primers used in the study

Primer	Primer sequence from 5’-3’
PRD37996.1-F	GCCACGCTGTAAGCCAAGACTTAG
PRD37996.1-R	CGAATCCGCAGTGCCGAATCC
RZB40889.1-F	CCAGCGAGCACCAACAAAATTACG
RZB40889.1-R	GCAACCGAGGAGTTCCAAGTGTG
XP_019759670.1-F	GGACCAAGATCCCTGCCCAGAC
XP_019759670.1-R	GCTTTCAAATTCTGCCAGGGTTGC
AZL87164.1-F	GCGTAATGGCCGATATGACACC
AZL87164.1-R	CCTTTCCTGGCTTTATCGACGG
XP_019870227.1-F	GACCGTCCAGTAATCCTCCTCCTC
XP_019870227.1-R	TGTCCGAATCGCCGAATCCTTTG
XP_025834808.1-F	GCAAACATCACGTTCACACCC
XP_025834808.1-R	TCACCAAGCAGTTTATCTCCGTT
XP_008193582.1-F	TTTGTTTGCCAGGGGAGTGTTTTG
XP_008193582.1-R	TGCAAAATTCGGCAATCCTTTGGC
NP_001164248.1-F	AATCCGCATAAGTGGATGCTGGTC
NP_001164248.1-R	GTACTCGGGAGCTAAGCCTTTGTG
KMQ88340.1-F	TGGCTTCTACTCCGACTGGTCTG
KMQ88340.1-R	GCCACCGACGCACACTTCTG
XP_008194340.1-F	ACAGCGCCTGATGGACAACA
XP_008194340.1-R	TCGCTGGAGGAATTGGAGGG
XP_028141204.1-F	CTGTTCACTACACCGCTGATGACC
XP_028141204.1-R	GAGCGTGTACAAGAGGAGCATGAG
KYM76060.1-F	GCCACCATTGACTTGCCATTGC
KYM76060.1-R	TGGATGATCGGCGTAACACTTGTC
XP_018334196.1-F	AACAAGAGCAAGAAGGCGTACTCC
XP_018334196.1-R	TCCGGTCTCGTCAGCGTAAAATTC
EFN71883.1-F	TCCACGGGTTATCCTTTACTTGGT
EFN71883.1-R	AGTACCGATCATCTGCCTGCA
Actin-F	CGTACCACCGGTATCGTATTG
Actin-R	CGGAGGATAGCATGAGGTAAAG

### Statistical analyses

SPSS 19.0 (for Windows; SPSS, Chicago, IL, USA) was used for all statistical analyses. One-way ANOVA at a significant level of P < 0.05 was used to compare data relating to longevity, oviposition of *S. japanicum* and survival rate, developmental duration and predation of first generation of *S. japanicum* under different treatments.

## Electronic supplementary material

Below is the link to the electronic supplementary material.

**Additional file** **1**.

**Additional file** **2**.

**Additional file 3.**

**Additional file 4.**

## Data Availability

The sequencing data generated in this study has been deposited to NCBI database under the NCBI accession number: PRJNA851894. The raw data sets generated for this study are available at: http://www.ncbi.nlm.nih.gov/bioproject/PRJNA851894 (NCBI accession number: SRP383169).
